# Exploring PPM1D Phosphatase Structure for Next-Generation Small Molecule Inhibitor Development

**DOI:** 10.1063/4.0001159

**Published:** 2025-10-27

**Authors:** Jay P. Kumar, Dalibor Kosek, Stewart Durell, Nadya I. Tarasova, Lisa Jenkins, Subrata Debnath, Nathan Coussens, Matthew Hall, Daniel Appella, Fred Dyda, Sharlyn Mazur, Ettore Appella

**Affiliations:** 1 National Cancer Institute, NIH, Bethesda, MD, USA; 2 NIDDK, National Institutes of Health, Bethesda, MD, USA; 3 Institute of Physiology of the Czech Academy of Sciences, Vestec, Prague, Czech Republic; 4 National Cancer Institute, NIH, Frederick, MD, USA; 5 National Center for Advancing Translational Sciences, NIH, Rockville, MD, USA

## Abstract

The Wild-type p53-induced phosphatase (Wip1, or PPM1D) is a serine/threonine phosphatase that plays a central role in regulating cellular stress response pathways, including p53 signaling and DNA damage repair. Its overexpression and gain-of-function mutations have been associated with various cancers, making it an emerging therapeutic target. However, efforts to develop selective inhibitors have been limited by a lack of structural information and the inherent challenges of targeting phosphatases, particularly due to the unique and flexible regions characteristic of PPM family members. Here, we report the first crystal structure of the PPM1D catalytic domain to 1.8 Å resolution. The structure provides insights into the active site with two bound Mg2+ ions. The flap subdomain and B-loop, crucial for substrate recognition and catalysis, were also resolved. Unexpectedly, we observed a nitrogen-oxygen-sulfur (NOS) covalent bridge within the crystal structure, which might contribute to structural stability, although its physiological relevance remains unclear. Molecular dynamics simulations and kinetic studies offered further mechanistic insights into the regulation of PPM1D catalytic activity. Leveraging these structural insights, we conducted a structure-based virtual screening of over 21 million compounds and identified the 9 most promising compounds with the highest potential for PPM1D inhibition. We are validating these lead compounds and developing analogs to enhance effectiveness. In summary, this work advances the understanding of PPM1D function and facilitates the further development of specific inhibitors of PPM1D phosphatase.

